# Supplementary figures and images for: Amyloidogenic and Neuroinflammatory Molecular Pathways Are Contrasted Using Menaquinone 4 (MK4) and Reduced Menaquinone 7 (MK7R) in Association with Increased DNA Methylation in SK-N-BE Neuroblastoma Cell Line
Source: Cells. 2023 Dec 27;13(1):58. doi: 10.3390/cells13010058 (PMC10778373; doi:10.3390/cells13010058)

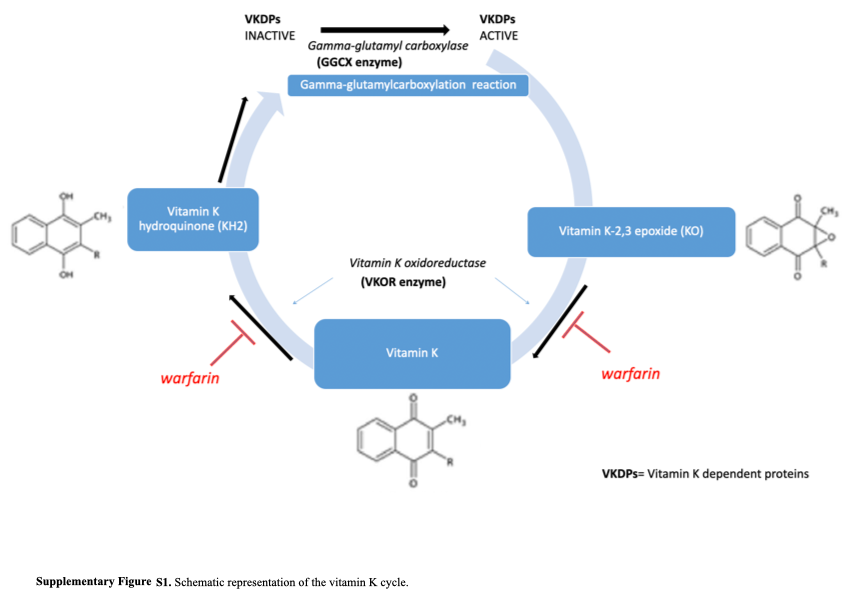

Supplement: Supplementary file 1 [file cells-13-00058-s001.zip › Figure S1.tiff]

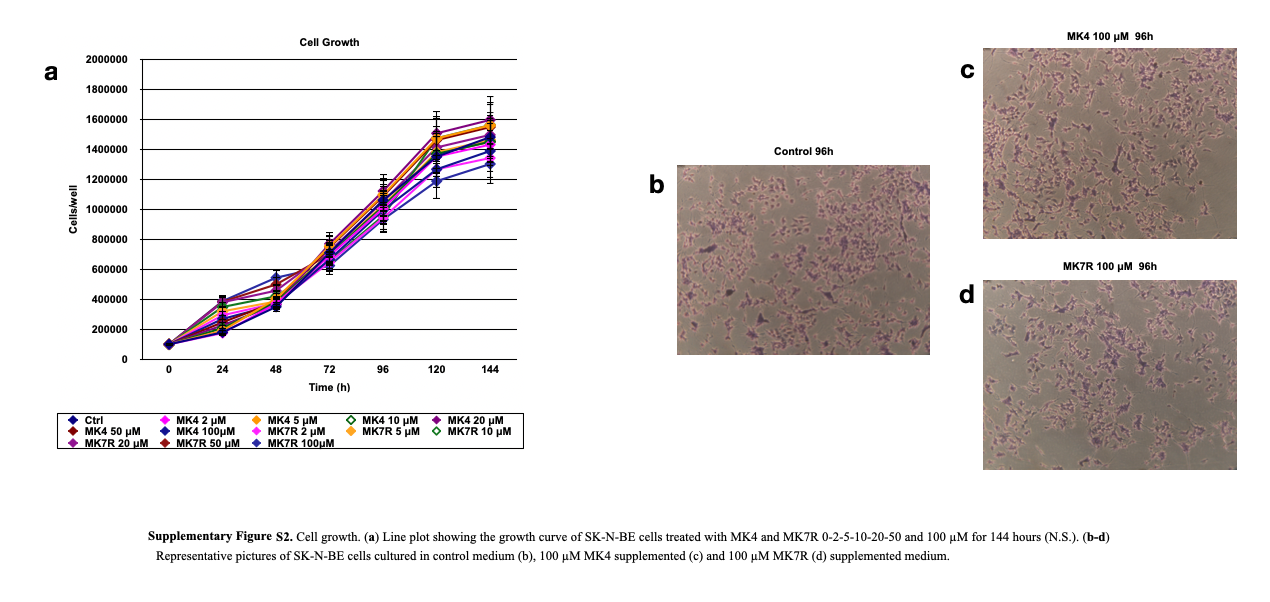

Supplement: Supplementary file 1 [file cells-13-00058-s001.zip › Figure S2.tiff]

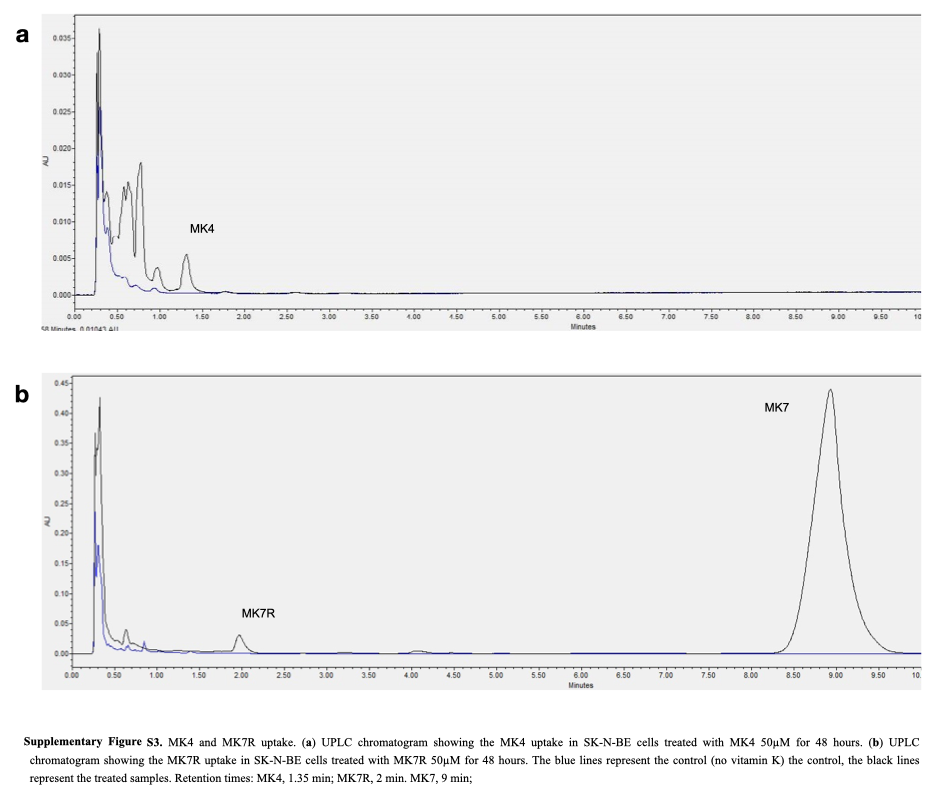

Supplement: Supplementary file 1 [file cells-13-00058-s001.zip › Figure S3.tiff]

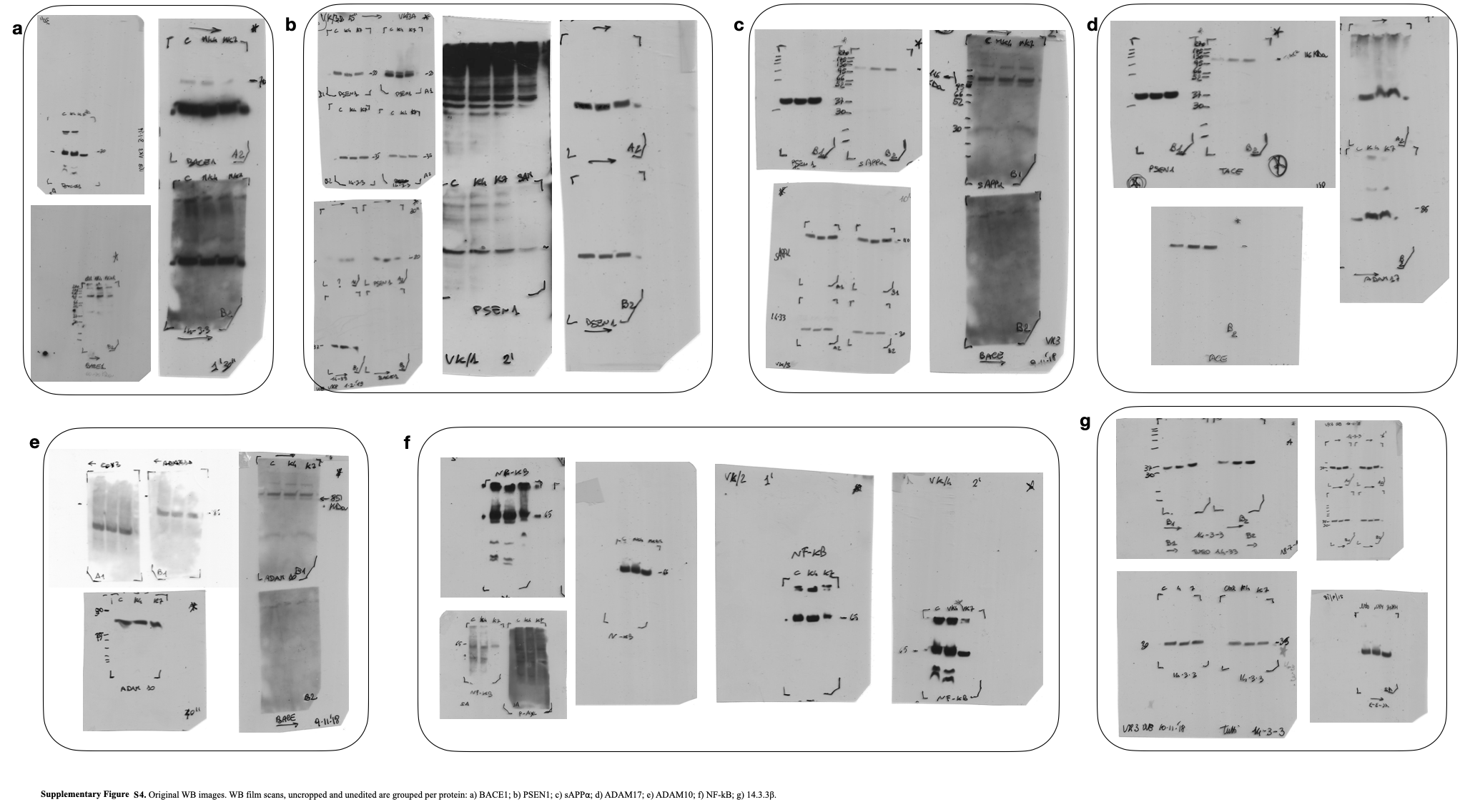

Supplement: Supplementary file 1 [file cells-13-00058-s001.zip › Figure S4.tiff]

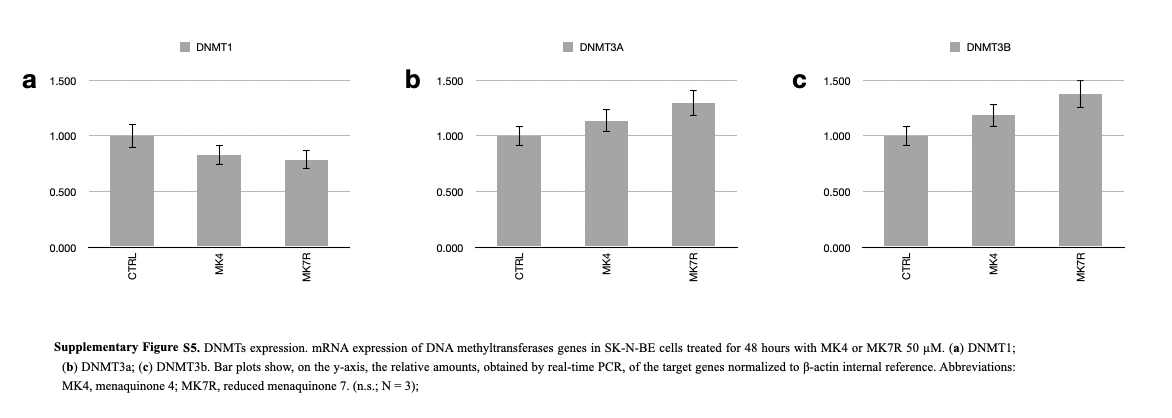

Supplement: Supplementary file 1 [file cells-13-00058-s001.zip › Figure S5.tiff]
